# Editorial: Unveiling immune biomarkers: advancing trauma care through cellular and vesicular insights

**DOI:** 10.3389/fimmu.2025.1716977

**Published:** 2025-10-17

**Authors:** Arnulf G. Willms, Aliona Wöhler, Robert Schwab, Sebastian Schaaf, Artur Slomka, Ionita Ghiran, Simon C. Robson, Miroslaw T. Kornek

**Affiliations:** ^1^ Department of General, Visceral and Thoracic Surgery, German Armed Forces Central Hospital, Koblenz, Germany; ^2^ Department of Pathophysiology, Nicolaus Copernicus University in Toruń, Ludwik Rydygier Collegium Medicum in Bydgoszcz, Bydgoszcz, Poland; ^3^ Department of Hematology and Oncology, National Medical Institute of the Ministry of Interior and Administration, Warsaw, Poland; ^4^ Center for Inflammation Research, Department of Anesthesia, Beth Israel Deaconess Medical Center, Harvard Medical School, Boston, MA, United States

**Keywords:** trauma, biomarker, immune system, macrophages, neutrophils, neurotrauma

The exploration of biomarkers in trauma and critical care is an evolving field, with cellular, molecular, and vesicular pathways offering insights into immune dysregulation after injury. In recent years, liquid biopsy–based approaches have emerged as promising tools, exemplified by initiatives such as LiBOD™, which explore circulating markers derived from diverse cell populations and tissues (1–9). These markers may be released directly in response to the traumatic insult, or indirectly through post-trauma processes such as inflammation, tissue remodeling, and wound healing. Such approaches underscore the potential of minimally invasive sampling to capture systemic responses in real time and to provide clinically relevant insights into injury progression and prognosis.

Despite fewer contributions than anticipated, the accepted papers in this Research Topic highlight both the diversity of approaches and the translational potential of biomarker research. Together, they underscore the complexity of immune responses in trauma and related conditions, while pointing to new directions for clinical and experimental investigation.

## Summary of contributions

1. Neutrophil monitoring in surgery: Jovanovski et al. investigated multimodal monitoring of neutrophil activity during cardiac surgery. Their findings revealed dynamic intraoperative changes in neutrophil function, which preceded conventional inflammatory markers such as CRP and IL-6. This work emphasizes the potential of early immune monitoring for predicting perioperative complications and tailoring interventions.

2. Exosomes and macrophages in sepsis-induced ALI: Lv and Liang reviewed the role of exosomes in modulating macrophage polarization during sepsis-induced acute lung injury. They discuss how exosomal cargo from pathogens, neutrophils, epithelial cells, and mesenchymal stromal cells influences macrophage phenotype, with implications for both injury progression and therapeutic strategies. Their perspective positions exosomes as promising targets for future immune-modulating therapies.

3. Bioinformatics discovery of a novel TBI biomarker: Ding et al. identified KDM5D as a novel biomarker for traumatic brain injury through bioinformatics and experimental validation. Combining transcriptomic analysis, machine learning, and validation in rat models, they demonstrated diagnostic potential for KDM5D. This study highlights how computational pipelines, integrated with preclinical models, can accelerate biomarker discovery in neurotrauma.

4. Extracellular vesicle proteomics in burn injury: Willis et al. characterized temporal changes in extracellular vesicle protein cargo after severe burn injury, using both human samples and mouse models. Their findings showed distinct immune reprogramming effects of EVs in early vs. late phases post-injury, suggesting a role for EV protein cargo as biomarkers of immune status and outcome in burn patients.

Although limited in number, the accepted manuscripts illustrate the wide methodological spectrum of trauma biomarker research — spanning clinical immunomonitoring, bioinformatics-driven discovery, mechanistic reviews, and EV proteomics. Together, they highlight three central themes:

- The immune system as a dynamic sensor: Neutrophils and macrophages rapidly integrate signals of injury and infection, shaping outcomes in ways not captured by classical biomarkers.
- Extracellular vesicles as versatile mediators: Both reviews and original research confirm their role in modulating inflammation and their potential as circulating, non-invasive biomarkers.
- The power of translational pipelines: Bioinformatics, preclinical validation, and clinical data integration are accelerating the identification of novel markers.

In sum, our Research Topic, underscores the importance of continued efforts to unravel the immune system’s role in trauma. Which still remains a wide frontier, and these contributions provide stepping stones toward a more precise, individualized management of critically injured patients. The four contributions highlight promising avenues — from neutrophil monitoring and macrophage reprogramming to novel gene markers and EV proteomics. Together, they provide distinct directions for future collaborative work aimed at translating immune biomarkers into clinical practice for trauma and critical care.
